# The effect of an organizational level participatory intervention in secondary vocational education on work-related health outcomes: results of a controlled trial

**DOI:** 10.1186/s12889-017-4057-6

**Published:** 2017-01-31

**Authors:** Roosmarijn M. C. Schelvis, Noortje M. Wiezer, Allard J. van der Beek, Jos W. R. Twisk, Ernst T. Bohlmeijer, Karen M. Oude Hengel

**Affiliations:** 1 Netherlands Organization for Applied Scientific Research TNO, Work, Health and Technology, P.O. Box 3005, NL-2301 DA Leiden, The Netherlands; 2 Body@Work, Research Center on Physical Activity, Work and Health, TNO-VUmc, P.O. Box 3005, NL-2301 DA Leiden, The Netherlands; 3 Department of Public and Occupational Health, the EMGO+ Institute for Health and Care Research, VU University Medical Center, P.O. Box 7057, NL-1007 MB Amsterdam, The Netherlands; 4 Department of Epidemiology and Biostatistics, VU University Medical Center, De Boelelaan 1089a, Amsterdam, NL-1081 HV The Netherlands; 5 Department of Health Sciences, VU University, Amsterdam, The Netherlands; 6 Department of Psychology, Health and Technology, University of Twente, P. O. Box 217, NL-7500 AE Enschede, The Netherlands

**Keywords:** Self-efficacy, Work-related stress, Well-being, Teacher

## Abstract

**Background:**

Work-related stress is highly prevalent in the educational sector. The aim of the current study was to evaluate the effectiveness of an organizational level, participatory intervention on need for recovery and vitality in educational workers. It was hypothesized that the intervention would decrease need for recovery and increase vitality.

**Methods:**

A quasi-experiment was conducted at two secondary Vocational Education and Training schools (*N* = 356) with 12- and 24-months follow-up measurements. The intervention consisted of 1) a needs assessment phase, wherein staff and teachers developed actions for happy and healthy working under supervision of a facilitator, and 2) an implementation phase, wherein these actions were implemented by the management teams. Mixed model analysis was applied in order to assess the differences between the intervention and control group on average over time. All analyses were corrected for baseline values and several covariates.

**Results:**

No effects of the intervention were found on need for recovery, vitality and most of the secondary outcomes. Two small, statistically significant effects were in unfavorable direction: the intervention group scored on average over time significantly lower on absorption (i.e. a subscale of work engagement) and organizational efficacy than the control group.

**Conclusions:**

Since no beneficial effects of this intervention were found on the primary and most of the secondary outcomes, further implementation of the intervention in its current form is not eligible. We recommend that future organizational level interventions for occupational health 1) incorporate an elaborate implementation strategy, 2) are more specific in relating actions to stressors in the context, and 3) are integrated with secondary preventive, individual focused stress management interventions.

**Trial registration:**

Netherlands Trial Register NTR3284 (date registered: February 14 2012).

## Background

Twenty percent of the EU workers consider their health to be at risk as a result of work-related stress [1]. Work-related stress is especially common among workers in education throughout both the eastern [2, 3] and western developed world [4]. According to a report for the Health & Safety Executive the stress levels of teachers were more than double (42%) compared to those in other occupations [5]. Also in the Netherlands, one in five employees suffer from work-related stress, according to a representative survey [6]. In secondary Vocational Education and Training (VET) this would equal to 11,174 of the currently employed 52,456 workers [7]. These workers feel emotionally drained and exhausted, especially at the end of the work day, and tired when they get up again in the morning [6]. For 6.9% of the workers in Dutch education, work-related stress results in being overworked or burned out, including long term sick leave [6].

Work-related stress may show in workers as decreased vitality and increased need for recovery, the primary outcomes of this study. High vitality is defined as having high levels of energy and resilience, persisting in the face of difficulties, and willingness to invest effort in the work [8]. Need for recovery is the extent to which employees experience problems in the recovery of efforts at work, and is hence indicative of early symptoms of fatigue at work [9]. Work-related stress and reduced well-being can result from an imbalance between two types of workplace characteristics: job demands and job resources. The physical, social or organizational aspects of the job that require sustained physical or psychological effort are considered job demands, whereas job resources are those work aspects that may reduce job demands, help to achieve goals and stimulate learning and development [10]. Over the years, job demands have intensified in the educational sector [11], while job resources remained the same. Examples of increasing job demands are the growth in accountability measures [12] or the integration of students with special needs in the regular classes [13]. Job demands and job resources have to be balanced in order to prevent stress [14, 15]. A job demand, such as dealing with students with special needs, will turn into a stressor over time if job resources, such as coworker support, are insufficient or lacking [16, 17]. The imbalance between demands and resources likely contributed to the current work-related stress prevalence.

An imbalance between job demands and job resources can be restored by primary preventive interventions, which aim to alter the source of the stress at work. However, an overview of stress management interventions showed that typical stress management interventions mostly aimed to reduce stress symptoms (i.e. secondary prevention) [18]. Moreover, the existing interventions were targeted at the individual level and comprised stress management and coping techniques [18], whereas a review demonstrated that the organizational level is to be preferred for implementing stress management interventions, because organizational level interventions are more likely to bring about positive changes than the individual level interventions [19]. To date, the interventions targeted at workers in education primarily aimed to enhance the individual capacity of (trainee) teachers or teaching assistants to cope with stressors in the workplace, for example via mindfulness-based stress reduction or workshops on stress management skills [20–24]. These interventions were only partly effective in influencing work-related stress or (dimensions of) burnout [20–24] and well-being [24].

An organizational level intervention focuses on changing stressors in the work environment, rather than changing the response of employees to stressors, and the change consists of altering some aspect of the organization (e.g. roles, structure) [25]. However, more is needed than just applying a primary preventive, organizational level intervention to render effective results [26, 27]. First, participation of stakeholders is acknowledged as one of the most desirable intervention strategies [28], since it leads to a feeling of joint ownership of both problems and solutions and thereby increases implementation and long term adherence. Secondly, self-efficacy beliefs of the target group are of importance for interventions targeted at changing the root-cause of stress [29]. Self-efficacy is ‘the belief in one’s own ability to master specific domains in order to produce given attainments’ [30, 31]. High self-efficacy would help workers to create a ‘control over circumstances mindset’ [32]. The most effective way to enhance self-efficacy is by providing a mastery experience, and it was assumed that taking part in the first phase of the intervention leads to this experience of mastery.

**Fig. 1:**
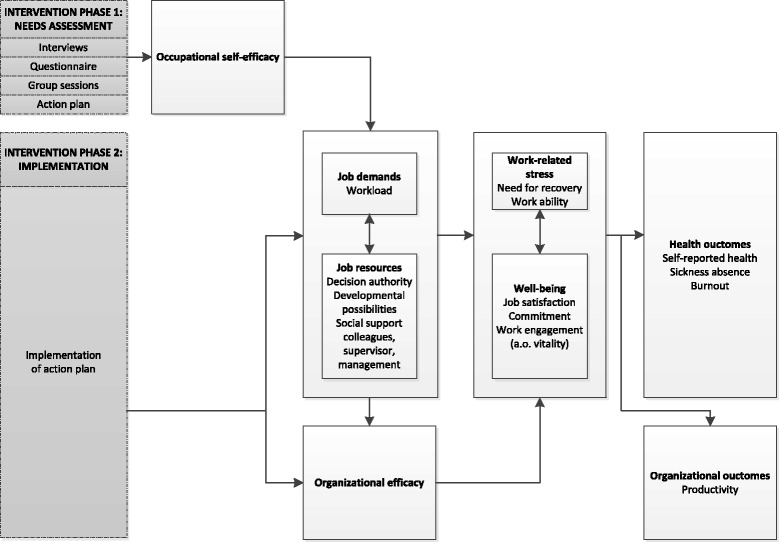
Model representing the logic order of expected changes

The aim of the current study was to evaluate the long term effectiveness of an organizational level, primary preventive, participatory intervention on need for recovery and vitality primarily. The hypothesized order of expected changes is that participating in the intervention’s first phase (needs assessment) would result directly in participant’s increased occupational self-efficacy (proximal effect, Fig. 1). Implementation of intervention activities (the intervention’s second phase) would increase organizational efficacy and job resources (i.e. decision authority, developmental possibilities and various forms of social support) and reduce job demands (i.e. psychological demands), these are the expected intermediate effects (Fig. 1). And if the balance between job demands and job resources is restored, distal effects are supposedly to be found on work-related stress constructs (i.e. need for recovery and work ability) and well-being constructs (i.e. work engagement including vitality, job satisfaction and commitment; Fig. 1).

## Methods

### Study design

The effectiveness of the intervention was evaluated in a controlled trial with a matched control group and three measurements (T0, T1, and T2). First follow-up measurement was conducted at 12 months after baseline (T1) and second follow-up at 24 months after baseline (T2). Data were collected via a questionnaire constructed with online survey software, participants received a link to the questionnaires in their mailbox. To increase the response rate at T1 and T2, an incentive (i.e. a warehouse gift card) was sent to respondents in the intervention and control group.

This trial was registered in the Netherlands Trial Register (NTR3284). The study has been approved by TNO’s Review Committee Participants in Experiments (RCPE), an internal ethics committee that assesses ethical aspects of working with participants in experiments. The RCPE advised positively on the study to the responsible manager since the committee perceived “the information to be complete, participants can join voluntarily and an informed consent is provided” [33]. The manager decided to follow the RCPE’s approval by permitting the study.

### Study population

Two Vocational Education and Training (VET) schools were recruited via a mailing by the sector organization, The Netherlands Association of VET Colleges. A high sickness absence rate within a certain department was the most important reason to participate in this study, according to the Executive Boards of both schools. Therefore these two departments were selected as intervention groups by the schools. According to the directors of departments that were selected as intervention groups, their concerns about the situation in their department and a notion of their employees’ diminishing happiness at work were important deliberations to participate. The researchers matched a control group within the same school to each intervention group, based on department size, mean age and type of work. In total, four departments were included. Since the intervention and control groups were situated in different locations we consider diffusion of treatment effects to be unlikely. All teaching and non-teaching (i.e. educational and administrative support staff) employees and their managers in these departments were invited to participate in the study. Employees who worked within the school, but did not teach at a secondary vocational level were excluded. Informed consent was obtained from all individual participants included in the study.

### Matching, blinding and sample size

In each school, one department was selected as the experimental group by the participating schools, since their motive to participate in this study was to solve a problem or reach a goal within a specific department. To reduce the negative impact of selection bias, the control groups were obtained according to the ‘general control’ matching principle [34] on the criteria: department size (at least 150 employees), age composition of staffing, and type of work (i.e. teaching vocational students and not secondary school pupils). Blinding of the participants and intervention providers was impossible due to the participatory nature of the intervention.

The sample size calculation was based on the number of cases required to detect an effect (Cohen’s d = 0.2) on the primary outcome vitality, as measured with the 3-item subscale ‘vigor’ of the Utrecht Work Engagement Scale-9 (UWES-9) [35]. With a power of 80%, a two-sided alpha of 5%, the required sample size is 385, which translates to 193 participants per school and 97 per group. With an expected loss to follow-up of 35% over 24 months, a total sample size of 600 was needed at baseline. The sample size calculation has been described extensively elsewhere [33].

### Intervention

The intervention was a participatory action approach applied at the organizational level, named the Heuristic Method (HM). HM was developed by a Dutch consultancy firm and piloted over a hundred times in public and private organizations before evaluation within this trial. The consultancy firm refined the intervention after each application, based on the lessons learned. Although the customers were almost always satisfied with the intervention’s results, the intervention effects were never tested scientifically. The intervention consisted of two 12-month phases: (i) a phase of needs assessment, and (ii) an implementation phase.

In the needs assessment phase, staff and teachers developed actions to ‘work happily and healthily’, under supervision of an HM facilitator. The HM facilitator held expertise in organizational change processes, and he used the management’s and workers’ knowledge, skills and perceptions to thoroughly determine what hindered and facilitated ‘healthy and happy working’ in the organization. A participatory work group was formed, its members were ambassadors of the project and assisted the facilitator (e.g. by approaching interviewees or by proof reading reports).

The HM facilitator then led three iterative steps to complete the needs assessment by: (i) approximately ten one-hour interviews with typical optimistic and typical critical teachers and staff; (ii) a digital open-ended questionnaire for all workers; and (iii) group sessions with all teams, chaired by members of the participatory group. The result of each step in the intervention determined the content of the following step. Reports of each step were written by the HM facilitator and were shared with all workers, starting with the participatory work group, then the management team, and finally with all workers. The third and last report contained the facilitator’s advice to the management team on intervention activities to be implemented in the next phase. Examples of these activities were: creating a staff room or implementing performance assessment policies (see Table 1 for an overview of all problems, goals and intervention activities).

**Table 1:** Results of the needs assessment and translation into action plan

	Main content of advisory report delivered by facilitator	Main content of action plan^a^ constructed by management team
School A	(i) professionalize the teams;	The director, assisted by an HM consultant, translated the recommendations into an action plan with three goals, six changes and a set of quick wins.
	(ii) professionalize the management;	Goals: i) unambiguous management control; ii) competence and professionalism in the teams, and iii) adequate facilities
	(iii) improve the administrative support and facilities.	Changes: (i) compliance to the workload policy, (ii) structured performance reviews; (iii) a continuous dialogue on the organization of the educational programs; (iv) a leading team activities plan; (v) weekly work meetings; and (vi) personalized competence development plans.
		Quick wins: create adequate facilities by creating a staff room at both locations; place extra walls in some classrooms; place beamers in all class rooms; improve the service by the facilitation services office.
School B	(i) create adequate and effective management control by installing a management team that is approachable, coaching, and leading;	The directors of the management team decided to integrate the facilitator’s recommendations in the annual agreements (i.e. a management contract) she made with the Executive Board, instead of writing a separate action plan. A coach was attracted to support teams in a previously initiated change towards becoming self-managing.
	(ii) make teams the central executive units by developing a team program;	Goals were formulated in four headlines: i) strategy; ii) education; iii) personnel; iv) organization; and v) business operations.
	(iii) eliminate cumbersome administrative procedures.	The most important change per headline was: i) alliances with partners in the region are closed; ii) the curriculum of two educations are reconstructed into units of learning; iii) performance review policies are implemented; iv) teams function as self-managing units; and v) a multi-annual housing plan is developed.
		No quick wins were formulated.

^a^Action plan was termed ‘Management Contract’ in school B

In the implementation phase, the intervention activities were implemented by the management teams at both schools. HM prescribed that the management team translated the facilitator’s advisory report into an action plan, containing an implementation plan, comprising at least a timeframe, a budget and the allocation of roles. Assistance by the HM facilitator could be provided if the management had the means to temporarily hire a consultant.

### Primary outcome measures

Primary outcomes were an indicator of work-related stress (i.e. need for recovery) and well-being (i.e. vitality).

#### Need for Recovery

The concept was assessed using a subscale of the Dutch Perception and Evaluation of Work Questionnaire [9]. The scale comprises 11 dichotomous (yes/no) statements such as “My job makes me feel rather exhausted at the end of a work day”. The need for recovery scale ranges from 0 to 100, calculated as the number of points (1 = yes, 0 = no) divided by the number of questions answered, multiplied by 100. Higher scores indicate a higher need for recovery, which is unfavorable. The questionnaire has proven to be valid and reliable (Cronbach’s alpha 0.86) [9]. In the current study, internal consistency was excellent (Cronbach’s alpha: 0.89).

#### Vitality

Vitality was assessed using the 3-item vigor subscale of the Utrecht Work Engagement Scale-9 (UWES-9; e.g. “At my job, I feel strong and vigorous”) [35]. Response scales range from 0 (never) to 6 (always/every day). The subscale scores were obtained by calculating the mean (range 0–6). Higher scores are indicative of more vigor. The total UWES-9 has shown good validity and reliability [36], as was the case for the subscale in this study (Cronbach’s alpha: 0.87).

### Secondary outcome measures

Several categories of secondary outcomes were measured: job demands, job resources, indicators of work-related stress, well-being and efficacy. Job demands were operationalized as psychological demands and job resources as decision authority, developmental possibilities and various forms of social support. Work-related stress was indicated as reduced work ability, well-being was indicated by work engagement, job satisfaction and commitment. Two efficacy or competence measures were taken into account: occupational self-efficacy and organizational efficacy.

#### Psychological demands

A five item subscale of the Dutch version of the Job Content Questionnaire (JCQ) was used to measure psychological demands, e.g. “My job requires that I work very fast”. Scale reliability and validity was acceptable upon construction [37], as was the case in the current study (Cronbach’s alpha: 0.68). The response scale ranged from 1 (strongly disagree) to 4 (strongly agree), and the scale score was calculated as the sum of the individual items (range 4–16) [37]. Higher scores indicate higher job demands, which is unfavorable.

#### Decision authority

The Dutch version of the Job Content Questionnaire (JCQ) was used to assess decision authority. The three item subscale comprised items such as “My job allows me to make many decisions myself”. Scale reliability and validity was acceptable upon construction [37], in the current study it was good (Cronbach’s alpha: 0.79). The response scale ranges from 1 (strongly disagree) to 4 (strongly agree). The scale score was obtained by summing all the individual items (range 3–12). Higher scores indicate higher decision authority, which is positive.

#### Developmental possibilities

This concept was assessed with a subscale of the Dutch Well-being Checklist for Education [38], comprising four items, for example: “My work gives me the opportunity to learn new things”. The scale has shown good reliability (alpha 0.87) [39]. In the current study, internal consistency was acceptable (Cronbach’s alpha: 0.77). The response scale ranged from 1 (strongly disagree) to 5 (strongly agree), and the summed scale score ranged from 4 to 20. The higher the scale score, the more developmental possibilities were perceived, which is favorable.

#### Social support

The social support of colleagues, supervisor and management was measured using a modified version of two subscales of the Dutch version of the Job Content Questionnaire (JCQ) [37]. Each of these three subscales comprises three items, such as “My colleagues/my supervisor/the management help(s) to get the job done”. In the current study, internal consistency of the respective scales was excellent (Cronbach’s alpha: 0.99; 0.98; and 0.98). The response scales range from 1 (strongly disagree) to 4 (strongly agree). A scale score was obtained by summing the three individual items (range 3–12). Higher scores are indicative of more social support, which is positive.

#### Work Ability

Work ability was measured using two of the seven dimensions of the Work Ability Index (WAI) [40]. Several studies have indicated that the first dimension, current work ability compared to lifetime best, could be used as an indicator of the status and progress of work ability [41, 42]. Reliability and validity of this scale have been shown to be adequate in a Dutch sample (Cronbach’s alpha 0.63 to 0.71) [43]. The scale comprises a question on perceived current work ability compared to lifetime best, measured on a frequency scale from 0 (unable to work) to 10 (very good). To additionally gain insight into work ability in relation to job demands, the second dimension of the WAI was added. This dimension comprises two questions on perceived work ability in relation to mental and physical job demands, recorded on a five-point frequency scale from 1 (very bad) to 5 (very good).

The combined scale score (range 2–20) was calculated as the sum of the score on current work ability and the weighted scores on the demands, according to the nature of the work. Higher scores indicate higher work ability, which is favorable. In the current study, internal consistency of the combined scale was good (Cronbach’s alpha: 0.76).

#### Job satisfaction

Two items of the Netherlands Working Conditions Survey 2010 [44] were measured to determine level of job satisfaction, namely: “to what extent are you, all things considered, satisfied with your job” and “[…], satisfied with your working conditions?” Response scales range from 1 (very dissatisfied) to 5 (very satisfied). The items were combined into one scale, showing an acceptable internal consistency (Cronbach’s alpha: 0.70). The scale score was calculated as the mean of the two items (range 1–5), with higher scores indicating higher satisfaction.

#### Commitment to work and the organization

This concept was assessed using five items of the Dutch NOVA-WEBA questionnaire [45, 46], such as “My work means a lot to me” and “I feel perfectly at home in this organization”. Response scales range from 1 (strongly disagree) to 5 (strongly agree), with the scale score calculated as the mean of the score of all five items (range 1–5). Higher mean scores indicate higher commitment. Validity and reliability were moderate in an earlier report (Cronbach’s alpha 0.68) [47]. In the current study, internal consistency was acceptable (Cronbach’s alpha: 0.73).

#### Work engagement

Work engagement was assessed using the Utrecht Work Engagement Scale-9 (UWES-9), with the 3-item subscales vigor (see primary outcome vitality), dedication (e.g. “I am proud of the work that I do”), and absorption (e.g. “I am immersed in my work”) [35]. Response scales range from 0 (never) to 6 (always/every day). The scale and subscale scores were obtained by calculating the mean (range 0–6). Higher scores are indicative of higher work engagement. UWES-9 has shown good validity and reliability [36], as was the case in the current study (Cronbach’s alpha: 0.87).

#### Occupational self-efficacy

A modified version of the short Occupational Self-Efficacy Scale, comprising six items, was used to measure occupational self-efficacy (e.g. “Whatever happens in my work, I can usually handle it” [48]. Internal consistency was excellent in the current study (Cronbach’s alpha: 0.85), as was the case in other studies (Cronbach’s alpha 0.85) [49]. The response scale ranged from 1 (strongly disagree) to 5 (strongly agree) and a scale score was obtained by summing all individual items (range 6–30). A higher score indicates higher self-efficacy, which is favorable.

#### Organizational efficacy

This concept was assessed using the Organizational Efficacy Scale, comprising seven items, e.g.: “To what extent do you think your organization is able to deliver services of the highest quality?” [50]. The questionnaire was valid and reliable in previous studies (alpha 0.81) [50], internal consistency was excellent (Cronbach’s alpha: 0.89) in the current study. The response scale ranges from 1 (strongly disagree) to 5 (strongly agree). A total scale score was obtained by summing all individual items (range 7–35), so that a higher scores indicates higher organizational efficacy, which is favorable. Contrary to all other measures, organizational efficacy was measured at first and second follow-up only.

#### Covariates

Data on potential effect modifiers or confounders were collected at baseline, including age (in years), gender (male, female), school location (one of 12 locations), highest level of education (secondary school, vocational, professional or academic), function (teacher, teaching assistant, support staff, or management staff), and job tenure (in years).

### Statistical analyses

All analyses were performed according to the intention to treat principle (i.e. the analyses are based on the initial treatment assignment), using IBM SPSS Statistics 22.

Baseline differences between the intervention and control group were checked by performing regression analyses for all outcomes and independent samples *t*-tests for all continuous variables and Pearson Chi-square tests for the dichotomous variable describing individual characteristics of the sample.

Selective attrition was checked by conducting loss to follow-up analyses. With independent samples *t*-tests, baseline scores of participants at first and/or second follow-up were compared to baseline scores of participants who did not fill out first and/or second follow-up (*p*-value < 0.05).

To assess the effect of the intervention, linear mixed models with a two level structure was used, i.e. repeated measures were clustered within workers. Mixed models are especially suitable for longitudinal datasets containing correlated and unbalanced data [51, 52]. For each outcome variable, a crude model was built (i.e. difference between intervention and control group on average over time, corrected for the baseline value of the outcome [53]) as well as an adjusted model (i.e. the crude model, including adjustment for possible confounders age, gender, school location, and educational level). For organizational efficacy data were gathered only at first and second follow-up, hence linear regression analyses were conducted adjusting for the score on first follow-up measurement and for possible confounders (i.e. age, gender, school location, and educational level).

Two additional analyses were performed. First, time and the interaction between group and time were added to the adjusted mixed model in order to investigate whether the intervention effect was different over time (with a *p*-value < .05 indicating an interaction effect). And secondly, we compared high compliers in phase 1 (participation in two or three of the intervention’s first phase elements) to the control group on the primary and secondary outcomes, while correcting for baseline values and covariates.

## Results

### Participant flow

**Fig. 2:**
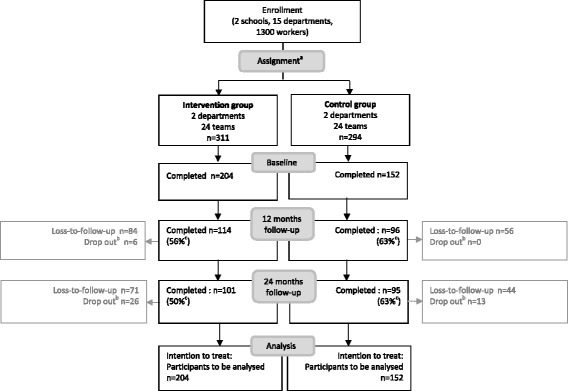
Flow diagram of the participants through the measurement moments of the trial. ^a^Assignment was based on matching criteria department size, age composition and type of work. ^b^The reason for drop out (i.e. discontinuing intervention) was in all cases termination of employment. ^c^Percentages are response percentages compared to baseline

The two schools were recruited in 2011. Figure 2 outlines the complete flow of participants through the study: of the 605 eligible workers from four departments, 356 (59%) completed the baseline questionnaire in February or June 2012. Between February 2013 and June 2014 the follow-up measurements were conducted. After 12 months, 210 participants completed the questionnaire (59%) and 6 participants dropped out due to termination of employment (Fig. 2). After 24 months, 196 participants (55%) completed the questionnaire and 39 dropped out due to termination of employment (Fig. 2). Following the intention to treat principle, the total number of employees to be analyzed is 204 for the intervention group and 152 for the control group (Fig. 2). Loss to follow-up analyses did not show any selective attrition of participants.

### Baseline characteristics

The baseline characteristics of the study population are presented in Table 2. Most of the teams were represented in the baseline sample (20 out of 24 in the intervention group, 21 out of 24 in the control group). Both the intervention and control group consisted mainly of highly educated workers (85.8 and 77.0%, respectively) and teachers (78.4 and 65.1%, respectively). However, the intervention group comprised more women, was of higher age, and had more years of service in education.

**Table 2:** Individual characteristics at baseline

	Total sample	Intervention group	Control group	*p*-value^a^
	*N* = 356	*N* = 204	*N* = 152	
Number of departments	4	2	2	-
Number of teams	41	20	21	-
Number of school locations	4	2	2	-
Gender (female) (%)	55.9%	65.2%	43.4%	.00*
Age (years)^b^ [mean (SD^c^)]	50.7 (9.2)	52.5 (8.5)	48.7 (9.5)	.01*
Tenure (years) [mean (SD^c^)]	18.3 (11.5)	20.3 (11.4)	15.6 (11.2)	.00*
Educational level (%)				.09
Secondary school	6.2%	5.4%	7.2%	
Vocational	11.8%	8.8%	15.8%	
Professional or academic	82.0%	85.8%	77.0%	
Function (%)				.03
Teacher	72.8%	78.4%	65.1%	
Teaching assistant	7.6%	4.9%	11.2%	
Support staff	13.2%	10.8%	16.4%	
Management staff	6.5%	5.9%	7.2%	

* *p*-value < 0.05

^a^Gender, education, and function tested with Chi-square test, age and tenure tested with an independent samples *t*-test

^b^Age based on *n* = 182 due to missings on this voluntary question

^c^SD is standard deviation

Table 3 shows the means and standard deviations of baseline measurements. Significant differences existed between the intervention and control group on most of the outcomes (except for secondary outcomes work ability, absorption, social support colleagues and supervisor), in favor of the control group (Table 3).

**Table 3:** Means and standard deviations at baseline, and at 12-month and 24-month follow-up

	Intervention Group		Control group		*p*-value^b^
	*n*	mean (SD^a^)	*n*	mean (SD^a^)	
Primary outcomes					
Need for recovery (0–100)					
Baseline	204	41.7 (33.6)	152	31.5 (30.7)	0.00*
12 months	112	47.5 (32.4)	92	36.1 (31.4)	
24 months	101	45.2 (33.5)	94	43.0 (33.0)	
Vitality (0–6)					
Baseline	204	4.2 (1.3)	152	4.5 (1.1)	0.00*
12 months	113	4.0 (1.3)	92	4.5 (0.9)	
24 months	101	4.1 (1.2)	95	4.3 (1.0)	
Secondary outcomes					
Psychological demands (4–16)					
Baseline	204	14.3 (2.2)	152	13.6 (2.0)	0.00*
12 months	114	14.2 (2.0)	96	13.6 (1.9)	
24 months	101	14.3 (2.5)	95	14.3 (1.9)	
Decision authority (3–12)					
Baseline	204	8.4 (1.4)	152	8.9 (1.5)	0.00*
12 months	114	8.0 (1.4)	96	8.7 (1.5)	
24 months	101	8.3 (1.5)	95	8.8 (1.4)	
Developmental possibilities (4–20)					
Baseline	204	13.3 (2.7)	152	14.1 (2.9)	0.00*
12 months	114	13.4 (2.6)	96	14.1 (2.6)	
24 months	101	13.6 (2.9)	95	14.2 (2.5)	
Social support colleagues (3–12)					
Baseline	204	9.3 (1.1)	152	9.3 (1.1)	0.96
12 months	111	9.0 (1.0)	91	9.1 (0.9)	
24 months	101	9.0 (0.8)	94	9.3 (1.1)	
Social support supervisor (3–12)					
Baseline	204	8.2 (1.5)	152	8.1 (1.7)	0.99
12 months	111	7.7 (1.6)	91	7.9 (1.6)	
24 months	101	7.9 (1.7)	94	7.6 (1.9)	
Social support management (3–12)					
Baseline	204	7.2 (1.7)	152	7.6 (1.6)	0.02*
12 months	111	6.8 (1.6)	91	7.2 (1.8)	
24 months	101	6.8 (2.0)	94	7.3 (1.8)	
Work ability (2–20)					
Baseline	204	15.3 (2.7)	152	15.9 (2.0)	0.02*
12 months	108	15.4 (2.3)	91	16.1 (2.1)	
24 months	99	15.3 (2.3)	91	15.4 (2.1)	
Job satisfaction (1–5)					
Baseline	204	3.3 (0.8)	152	3.7 (0.7)	0.00*
12 months	107	3.5 (0.7)	90	3.8 (0.7)	
24 months	99	3.3 (0.8)	91	3.6 (0.7)	
Commitment (1–5)					
Baseline	204	3.6 (.5)	152	3.8 (0.5)	0.00*
12 months	111	3.6 (.5)	90	3.8 (0.6)	
24 months	101	3.4 (.7)	94	3.8 (0.5)	
Work engagement (0–6)					
Baseline	204	4.0 (1.2)	152	4.3 (1.0)	0.00*
12 months	113	3.9 (1.2)	92	4.4 (0.9)	
24 months	101	3.9 (1.2)	95	4.2 (1.0)	
Dedication					
Baseline	204	4.1 (1.3)	152	4.6 (1.1)	0.00*
12 months	113	4.1 (1.3)	92	4.6 (0.9)	
24 months	101	4.1 (1.4)	95	4.5 (1.0)	
Absorption					
Baseline	204	3.7 (1.4)	152	3.9 (1.2)	0.00*
12 months	113	3.7 (1.4)	92	4.0 (1.1)	
24 months	101	3.6 (1.4)	95	3.9 (1.1)	
Occupational self-efficacy (5–30)					
Baseline	204	23.5 (3.2)	152	23.9 (2.7)	0.02*
12 months	113	22.5 (3.0)	92	22.8 (3.1)	
24 months	101	23.0 (3.4)	95	22.9 (2.9)	
Organizational efficacy^b^ (7–35)					
12 months	111	19.8 (4.8)	91	22.1 (4.6)	
24 months	101	19.7 (4.8)	94	22.0 (4.9)	

* *p*-value < 0.05

^a^SD is standard deviation

^b^All variables are tested with a regression analysis corrected for school

^c^Not measured at baseline

### Effectiveness of the intervention

The intervention effects on primary and secondary outcomes are presented in Table 4. No significant intervention effects were found on the primary outcomes need for recovery (the difference between the groups on average over time β = −3.2; 95% CI −12.1; 5.7) and vitality (β = 0.1; 95% CI −0.3; 0.4). For most of the secondary outcomes no intervention effect was found either, except for absorption (a subscale of work engagement) and organizational efficacy. For absorption, a significant intervention effect in unfavorable direction was found. The intervention group scored on average over time significantly lower on absorption than the control group (β = −0.3; 95% CI −0.6; −0.0). For organizational efficacy, a significant effect in unfavorable direction was found. The intervention group scored on average over time, significantly lower on organizational efficacy than the control group (β = −2.2; 95% CI −3.9; −0.5).

**Table 4:** Intervention effects on primary and secondary outcomes

	Crude model			Adjusted model^b^		
	Regression coefficient^a^	95% CI	*p*-value	Regression coefficient	95% CI	*p*-value^c^
Primary outcomes						
Need for recovery (0–100)	−0.486	−6.182; 5.209	0.867	−3.170	−12.067; 5.726	0.482
Vitality (0–6)	−0.010	−0.221; 0.200	0.922	0.059	−0.250; 0.368	0.707
Secondary outcomes						
Psychological demands (4–16)	0.016	−0.396; 0.428	0.939	−0.133	−0.668; 0.403	0.625
Decision authority (3–12)	−0.262	−0.544; 0.021	0.070	0.025	−0.387; 0.437	0.904
Developmental possibilities (6–30)	−0.432	−1.004; 0.141	0.139	−0.445	−1.339; 0.447	0.325
Social support colleagues (3–12)	−0.174	−0.365; 0.017	0.074	−0.156	−0.417; 0.103	0.236
Social support supervisor (3–12)	0.068	−0.278; 0.415	0.699	0.020	−0.484; 0.524	0.938
Social support management (3–12)	−0.259	−0.633; 0.115	0.174	−0.357	−0.834; 0.120	0.141
Work ability (1–10)	−0.173	−0.627; 0.280	0.452	0.134	−0.492; 0.761	0.672
Job satisfaction (1–5)	−0.124	−0.279; 0.030	0.115	−0.148	−0.366; 0.070	0.183
Commitment (1–5)	−0.151	−0.271; 0.032	0.013*	−0.163	−0.332; 0.006	0.058
Work engagement (0–6)	−0.037	−0.227; 0.154	0.706	−0.099	−0.360; 0.162	0.453
Dedication (0–6)	−0.055	−0.279; 0.169	0.629	−0.172	−0.471; 0.125	0.254
Absorption (0–6)	−0.132	−0.343; 0.078	0.216	−0.288	−0.576; −0.001	0.049*
Occupational self-efficacy (5–30)	0.149	−0.466; 0.763	0.634	0.065	−0.855; 0.985	0.889
Organizational efficacy^c^ (7–35)	0.165	−1.055; 1.386	0.790	−2.21	−3.906; −0.507	0.012*

Note. The correlation of repeated measurements within the individual (the personal ID level) is taken into account in the mixed model analyses. The clustering effect of workplaces/teams is taken into account by correcting for school location, by adding three dummy variables to the model

* *p*-value < 0.05

^a^The regression coefficient indicates the difference between the intervention and the control group on average over time, corrected for baseline value of the particular outcome

^b^Adjusted for age, gender, school location, and education level. The correlation of repeated measurements within the individual (the personal ID level) is taken into account in the mixed model analyses

^c^Measured for the first time at T1; regression coefficient is an unstandardized B

Significant interactions between group and time (i.e. effect of the intervention from baseline to T1) were observed on the primary outcomes need for recovery (*p* = .036) and vitality (*p* = .018) and the secondary outcomes social support of supervisor (*p* = .048) and work ability (*p* = .013). The interaction for need for recovery was negative (β = −10.97; 95% CI −21.91; −.74), whereas positive interactions were found for vitality (β = .44; 95% CI .07; .81), social support supervisor (β = .56; 95% CI .01; 1.11) and workability (β = 1.12; 95% CI .24; 2.00). This means that the ‘effects’ for need for recovery, vitality, social support of supervisor and work ability are stronger on T1 than on average over time. On the second additional analysis one effect was found: the high compliers scored on average over time significantly higher (*p* = .00) on occupational self-efficacy than the control group (β = 1.24; 95% CI 0.06; 2.42).

## Discussion

The current study aimed to evaluate the long term effectiveness of an organizational level, primary preventive, participatory intervention on need for recovery and vitality. Contrary to the hypothesis, the results showed no effects of the intervention on the aforementioned primary outcomes. For most secondary outcomes no effects were found either. However, statistically significant effects on two of the secondary outcome measures were in unfavorable direction (i.e. absorption as a subscale of work engagement, and organizational efficacy).

At least four aspects of the current study could explain the lack of effect. Firstly, we measured a wide range of positive and negative outcomes, but all measures were collected at the individual level. One could argue that an organizational level intervention requires organizational level collection of data to detect an effect, such as sickness absence registrations [27], team performance indicators or company results. A second reason regarding the type of outcomes could be that we defined and operationalized the outcomes before the trial. However, the exact type, content and implementation of actions was developed during the intervention. Therefore, the relation between actions taken and measures was possibly too distant to detect an effect. Third, the process evaluation demonstrated that implementation of the intervention’s first phase (needs assessment) was rather good, whereas the implementation of the actual changes in phase two (implementation phase) was poor in both schools [54]. Based on the level of implementation we expected to notice effects directly after intervention phase 1, at first follow-up, but these effects were only found for need for recovery, vitality, social support of supervisor and work ability. This finding should be interpreted with caution though, because it might as well be explained by a ‘ceiling effect’ in the high scores of the intervention group at baseline. For example, the baseline score of the intervention group for ‘need for recovery’ was 41.7. This is not only almost ten points higher than the control group, it is also higher than the mean score of around 30 points found in other studies (e.g. [9, 55, 56]). Such a high score at baseline makes an increase not likely. The fact that the improvement at T1 was not found for all outcomes might be due to the medium to low levels of satisfaction with the intervention. Hence, the lack of effect could be due to implementation failure. In post hoc analyses we tested for implementation failure and the effect found on occupational self-efficacy suggests that if the intervention would have been implemented as planned and the dose received would have been high enough for all, participants indeed might get a mastery experience out of taking part. Which in turn might lead to an increase in occupational self-efficacy. However, to reach this high dose received, the intervention’s implementation strategy ought to be revised so to ensure participation throughout both phases of the intervention (e.g. by planning all intervention elements during working hours). Fourth, the lack of effectiveness could be due to theory failure, it could have been that the theory behind the intervention did not address the problem righteously. In future participatory intervention studies researchers could consider constructs that are ‘closer’ to the actual implementation process as outcomes (e.g. participation, readiness for change).

### Comparison with earlier studies

Although the (partial) lack of effect was contrary to our expectations it is in line with some existing evidence on organizational-level interventions in education. A recent Cochrane review of organizational level interventions in (primary and secondary) education found only low-quality evidence that organizational interventions lead to improvements in teacher well-being and retention rates [57]. Low quality could for example be due to small numbers of participants or a lacking control group. However, the review included only four studies and in two cases teacher well-being was measured as a side effect of a student’s intervention, limiting the generalizability of the review’s outcomes.

The low or mediocre quality of evidence for organizational level interventions was also found in studies conducted outside of the educational domain. For example, the review by Montano and colleagues [58] included studies in health care, manufacturing and civil service mainly. The review demonstrated that comprehensive interventions, simultaneously addressing material, organizational, and work-time conditions, were more successful than single interventions. As a second example, an elaborate Cochrane review of stress management interventions of any type, conducted in health care, demonstrated that of the organizational-level interventions only changing work schedules may lead to a reduction of stress [59].

The current study adds to the existing body of evidence on the (partial) ineffectiveness of organizational level interventions for employee health. The evidence is considered to be relatively strong, since the design, with three measurements, was longitudinal as recommended in Michie and Williams’ review [60] and followed participants for a longer period than in most studies [57]. Secondly, a complex intervention framework was used as recommended for this target group and these outcomes [57]. Thirdly, validated measures were used for the operationalization of the concepts. And lastly, the theoretical concepts focused both on positive and negative work-related aspects, hence health protective and health promotive effects could be detected.

### Limitations of the current study

Some limitations of the current study need to be considered before generalizing the findings. Firstly, as a result of the long follow-up period of 12 and 24 months, loss to follow-up and drop out due to the termination of employment contracts were quite high. This probably affected the statistical power to detect changes. Secondly, although the matching was performed as effectively as possible, significant differences between the intervention and control groups persisted at baseline. This group difference was dealt with by correcting for baseline differences in all analyses [53]. A related, third limitation is the lack of randomization in this controlled trial: unknown confounding variables could be unevenly distributed over groups, threatening the internal validity. As has been described in the literature as a common challenge, the schools wanted to participate under the condition of choosing the intervention group [61]. Future studies of this type could consider alternative designs, such as the stepped wedge approach for selecting the order of groups receiving treatment, or methods, such as propensity score matching, to overcome the possible bias resulting from non-randomization [61].

### Recommendations for future research and practice

As described above, the current study already met some of the most important recommendations that were based on reviews of organizational level interventions and still no effects were found. There are at least three ways to further improve organizational level interventions. Montano and colleagues (2014) point to optimization of the implementation process as the strategy towards successful organizational-level interventions. The current study was conducted in daily practice and implementation suffered in this environment. The implementation is of utmost importance, since determinants of successful intervention [62] overlap with determinants of work-related stress (e.g. such as participation in decision making). By not implementing correctly, the facilitator or researcher could actually be adding a stressor to the work environment. The implementation strategy of this intervention should thus be revised (e.g. [63]) before the intervention can be recommended.

A second manner to improve effectiveness of organizational level interventions has been suggested by Ruotsalainen and colleagues: the interventions need to be more specific in their focus on stressors in order to be more effective [59]. In the current study the link between stressors formulated by all workers in phase 1 and actions taken by the management in phase 2 was unclear to most workers, which possibly hindered the effectiveness. This could be prevented by redesigning the implementation strategy in this intervention so as to incorporate participation as a central element in phase 2 as well, instead of leaving phase 2 to the management team in the schools.

Thirdly, organizational level interventions focusing on the root-cause of stress could be integrated with secondary preventive, individual focused stress management interventions. This simultaneous intervention on root-cause and early symptoms, could create a feeling of shared responsibility between organization and individual employee for occupational health. An integrated approach to workplace mental health might be more effective than one of the prevention types or levels alone [64].

## Conclusions

To our knowledge, this is one of the first primary preventive, organizational level intervention studies targeted at all workers in education. Reviews have shown the potential of this type of intervention [26, 27], especially if participation and mastery experiences are incorporated in the intervention strategy [28, 29]. Until now intervention studies that aimed to improve teacher well-being were secondary preventive and targeted at the individual level mostly [20, 21]. Unique is the content of the intervention; we evaluated a practice-based intervention that had been applied and redesigned over a hundred times for differing organizations, according to the consultancy firm which developed the intervention. However, the results of this evaluation showed no effects of this type of intervention on the primary outcomes. Two small, statistically significant effects on secondary outcomes absorption and organizational efficacy appeared to be in unfavorable direction. Post-hoc analyses showed that high compliers with the first phase of the intervention, scored on average over time significantly higher on occupational self-efficacy than the control group. Suggesting that if the ‘dose’ is high enough (i.e. implementation is sufficient), the intervention might offer participants a mastery experience which affects occupational self-efficacy. The intervention program in its current form lacks a sufficient implementation strategy and is therefore not recommended. Organizational level participatory interventions for occupational health should incorporate an elaborate implementation strategy and be more specific in relating the actions taken to the stressors in the context. Future intervention studies aiming to improve occupational health should consider integrating organizational level, primary preventive elements with individual, secondary preventive elements, in order to be effective [64].

## Abbreviations

HM: Heuristic method (i.e. the intervention)
JCQ: Job content questionnaire
NOVA-WEBA: Dutch questionnaire developed to identify risk factors for work stress
RCPE: Review committee participants in experiments
T0: Baseline measurement
T1: First follow-up measurement
T2: Second follow-up measurement
UWES-9: Utrecht work engagement scale-9
VET: Vocational Education and Training school
WAI: Work ability index.
